# Antioxidant and Anti-Inflammatory Effect and Probiotic Properties of Lactic Acid Bacteria Isolated from Canine and Feline Feces

**DOI:** 10.3390/microorganisms9091971

**Published:** 2021-09-16

**Authors:** Ki-Tae Kim, Jin-Woo Kim, Sun-Il Kim, Seonyoung Kim, Trung Hau Nguyen, Chang-Ho Kang

**Affiliations:** MEDIOGEN, Co., Ltd., Bio Valley 1-ro, Jecheon-si 27159, Korea; rlxo357@naver.com (K.-T.K.); valdicava@naver.com (J.-W.K.); sunil_2003@naver.com (S.-I.K.); clsrn3423@naver.com (S.K.); hau2807@naver.com (T.H.N.)

**Keywords:** lactic acid bacteria, antioxidant, anti-inflammatory, canine, feline

## Abstract

Oxidative stress is a phenomenon caused by an imbalance between the production of reactive oxygen species and antioxidant defenses. It plays an important role in numerous disease states, including chronic kidney disease, neurological disorders, cardiovascular diseases, diabetes, and cancer. Lactic acid bacteria (LAB) are known to have prominent antioxidant properties. Therefore, this study aimed to measure the antioxidant activity and anti-inflammatory potential of LAB isolated from animals for the efficient use of probiotics with host specificity. Antioxidant activity measurements of sixteen strains revealed that ABTS radical scavenging activities ranged from 26.3 to 57.4%, and DPPH free radical scavenging activities ranged from 4.7 to 13.5%. Based on the antioxidant activity assessment, five strains (*Enterococcus faecium* MG9003(YH9003)*, Enterococcus faecium* MG9007(YH9007), *Lactobacillus reuteri* MG9012(YH9012), *Lactobacillus fermentum* MG9014(YH9014), and *Pediococcus pentosaceus* MG9015(YH9015)) were selected with the consideration of fermentation productivity (>1 × 10^9^ CFU/g). The selected strains exhibited nitric oxide inhibition and inhibited inducible nitric oxide synthase and cyclooxygenase expression. Furthermore, probiotic properties, including intestinal adhesion and stability, were identified. Our results show that the selected animal-derived strains can be effective probiotic candidates for potential effects on animal hosts.

## 1. Introduction

Probiotics are live microorganisms with a positive influence on the balance of gastrointestinal microorganisms of their host when ingested in appropriate amounts [1]. They act as antioxidants through the ability to chelate metal ion, the generation of metabolites such as lactate, downregulation of reactive oxygen species (ROS)-producing enzymes, and upregulation of antioxidant activities [2]. These mechanisms improve the host’s defense against oxidative stress and contribute to the prevention of various diseases, including digestive disorders and cancer [3].

Oxidative stress is caused by excessive ROS, and the chemical reaction of oxygen radicals can cause irreversible cell and tissue damage [4]. In the majority of living organisms, ROS are eliminated through enzymatic and non-enzymatic defense mechanisms and repair systems to maintain homeostasis against oxidative stress [5,6]. Despite these inherent antioxidant defense systems, an imbalance in ROS production and antioxidant capabilities causes cell and tissue damage. Moreover, ROS-mediated mitogen-activated protein kinase (MAPK) signaling pathway activation is known to induce inflammatory cytokine production [7]. These reactions involve the expression of transcription factors, such as nuclear factor-κB (NF-κB), which play a key role in the synthesis of inflammatory proteins, such as nitric oxide synthase (NOS) and cyclooxygenase (COX-2) [8]. COX-2 is an enzyme involved in converting arachidonic acid from phospholipids in the cell membrane to prostaglandin H2 [9]. Continuous COX-2 overexpression is known to cause cancer and cardiovascular diseases. NOS is classified as neuronal NOS, endothelial NOS, and inducible NOS (iNOS). NO production by iNOS is high, and overproduction causes tissue damage and genetic variation [10].

In animal nutrition, the ban on antimicrobial growth promoters has focused on antibiotic replacement development, and studies have been conducted to assess the efficacy of host-protective probiotic feed additives to increase the productivity of various food-producing animals [11]. Additionally, as interest in promoting pet health increases, probiotic products for canines and felines are gaining popularity among owners. The microorganisms in probiotics exhibit host specificity, and successful probiotic use requires the use of strains derived from the same host [12]. Therefore, the purpose of this study was to examine the in vitro antioxidant and anti-inflammatory activities of lactic acid bacteria (LAB) isolated from canines and felines. Additionally, probiotic properties were evaluated for potential probiotic use in animals.

## 2. Materials and Methods

### 2.1. Isolation and Enumeration of LAB from Canines and Felines

Feces were collected from one dog (aged about 3 years, 15.8 kg body weight) and three cats (aged about 4 years, 4.3 (SEM 0.3) kg average body weight, male to female ratio of 2:1). All dogs and cats were clinically healthy and privately owned. We added 9 mL of buffered peptone water (BPW; Oxoid, Basingstoke, UK) to 1 g of fecal sample and mixed it using a Stomacher (3M, St. Paul, MN, USA). The mixture was serially diluted with BPW and plated on Rogosa agar (Difco, Detroit, MI, USA). To indirectly determine whether the isolates produced lactic acid, they were plated on de Man Rogosa Sharpe (MRS) agar containing 0.1% bromocresol purple. Only yellow colonies were selected and stored at −70 °C in 25% glycerol.

### 2.2. Identification of Isolated Bacterial Strains and Morphology

Identification of the isolated strains was performed by 16S rRNA gene sequencing via gene amplification using universal rRNA primers (27F, 1492R). Each process was carried out by SolGent (Daejeon, Korea). Sequences registered in the GenBank database were compared with the 16S rRNA sequence analysis results using the Basic Local Alignment Search Tool within the National Center for Biotechnology Institute (Bethesda, MD, USA). To identify the morphological properties of the isolated strains, 1% glutaraldehyde solution (Sigma-Aldrich, St. Louis, MO, USA) was used for immobilization at 4 °C for 24 h. The samples were dehydrated using ethanol and observed using a field emission scanning electron microscope (SEM; S4300, Hitachi, Tokyo, Japan).

### 2.3. Preparation of Cell-Free Supernatant

The cell-free supernatants (CFSs) of isolated LAB were prepared by modifying the method described by Lin et al. [2]. The isolated LAB strains were inoculated at an inoculum size of 2% (*v*/*v*) in MRS broth and cultured at 37 °C for 18 h. After 18 h of incubation, the bacterial suspension was centrifuged at 4000× *g* for 10 min at 4 °C and filtrated using 0.22 μm syringe filters (Millipore Co., Bedford, MA, USA).

### 2.4. Cell Culture Condition

RAW 264.7 cells were purchased from the Korean Cell Line Bank (Seoul, Korea). These cells were grown at 37 °C in 5% CO_2_ in fully humidified air. For routine subcultivation, Dulbecco’s modified Eagle’s medium (Gibco, Grand Island, NY, USA) supplemented with 10% fetal bovine serum (Gibco) and 1% penicillin–streptomycin (Gibco) was used and subcultured every 2 days to 80–90% confluence.

### 2.5. In Vitro Analysis of the Antioxidant Activity of Isolated Strains

#### 2.5.1. Scavenging Analysis of 2, 2′-Azino-bis(3–ethylbenzthiazoline–6-sulfonic Acid) (ABTS) Radical

The scavenging activity of ABTS radicals was measured following the method of Re et al. [13], with slight modifications. ABTS was dissolved in water to a concentration of 7 mM. The radical cation was generated by mixing ABTS stock solution with 2.45 mM potassium persulfate (1:1 *v*/*v*) and kept in the dark at room temperature for 24 h. Following incubation, the radical solution was further diluted with distilled water to an absorbance value of 0.7 ± 0.01 at 734 nm. The isolated LAB strains were incubated in MRS broth at 37 °C for 18 h and washed twice with phosphate buffer saline (PBS, pH 7.0). The absorbance was adjusted to 1.0, at a wavelength of 600 nm, to standardize the number of bacteria (10^7^–10^8^ CFU/mL), which were mixed with ABTS reagent at a 1:2 (*v*/*v*) ratio. Then, the solution was left at room temperature in the dark for 10 min and centrifuged. The control reaction was performed by adding PBS to ABTS solution. The absorbance of the mixture was measured at 734 nm. Each sample assay was performed in triplicate. The scavenging rate was calculated using the following equation:

ABTS radical scavenging activity (%) = (Ac − As)/Ac × 100(1)

where As is the absorbance of the test sample, and Ac is the absorbance of the control at 734 nm.

#### 2.5.2. Scavenging Analysis of 2,2-Diphenyl-1-picrylhydrazyl (DPPH) Radical

The scavenging of DPPH radicals was measured as described by Blois et al. [14]. After incubating the isolated LAB strains in MRS broth at 37 °C for 18 h, it was washed twice with PBS (pH 7.0). The absorbance was adjusted to 1.0, at a wavelength of 600 nm, to standardize the number of bacteria (10^7^–10^8^ CFU/mL), which were mixed with 0.05 mM DPPH reagent at a 1:2 (*v*/*v*) ratio. Then, the solution was left at room temperature in the dark for 30 min and centrifuged. The absorbance of each mixture was measured at 517 nm. Each sample assay was performed in triplicate. The antioxidant activity was calculated using the following formula:

DPPH radical scavenging activity (%) = (Ac − As)/Ac × 100(2)

where As is the absorbance of the test sample, and Ac is the absorbance of the control at 517 nm.

### 2.6. In Vitro Evaluation of Anti-Inflammatory Activities of Selected Strains

#### 2.6.1. NO Production in Murine Macrophage RAW 264.7 Cells

The NO release from RAW 264.7 cells was detected by measuring the NO_2_^−^ concentration, an indicator of NO synthesis; RAW 264.7 cells were seeded at 2 × 10^5^ cells/well in 96-well flat bottom microtiter plates (Corning, Corning, NY, USA) and stimulated with 0.5 μg/mL lipopolysaccharide (LPS), followed by the addition of CFSs (10%/well), and they were then incubated at 37 °C and 5% CO_2_. After 24 h of incubation, the culture supernatant and Griess reagent mixture was reacted at room temperature for 10 min [15], and the absorbance was measured at 550 nm to determine the NO concentration.

#### 2.6.2. RNA Extraction and Reverse Transcription–Polymerase Chain Reaction

Reverse transcription–polymerase chain reaction (RT-PCR) was performed to determine the iNOS and COX-2 mRNA expression. Total RNA was extracted from RAW 264.7 cells using PureLink™ RNA Mini Kit (Invitrogen, Carlsbad, CA, USA) according to the manufacturer’s recommendations. Equal RNA amounts were mixed with reverse transcriptase premix (Elpis Biotech, Daejeon, Korea) to synthesize cDNA. Reverse transcription was performed as follows: initiation for 60 min at 42 °C and termination for 5 min at 94 °C. cDNA was amplified using specific primers (Macrogen, Seoul, Korea). The MiniAmp™ Plus Thermal Cycler (Applied Biosystems, Foster City, CA, USA) was used with the following procedure: pre-denaturation for 5 min at 95 °C, denaturation for 45 s at 95 °C, annealing for 45 s at 60 °C (COX-2, glyceraldehydes-3-phosphate dehydrogenase (GAPDH)) or 63 °C (iNOS), extension for 1 min at 72 °C, and final extension for 5 min at 72 °C. The PCR products were stained with Loading STAR (Dyne Bio, Seoul, Korea) and electrophoresed on a 2.0% agarose gel. The quantity of each band intensity was calculated using Image J software and normalized to the amount of the GAPDH housekeeping gene.

### 2.7. Functional Characterization of Selected Strains as Potential Probiotics

#### 2.7.1. Tolerance to Simulated Gastrointestinal Tract Conditions

Resistance to stimulated gastrointestinal conditions was determined using the methods described by Maragkoudakis et al. [16]. After 18 h of incubation, the cells were centrifuged (3470× *g* for 10 min) and washed twice with PBS (pH 7.0). Cells were suspended in simulated gastric fluid (pH 3 and 4; adjusted with 1 N HCl) containing 3 g/L of pepsin (Sigma-Aldrich, St. Louis, MO, USA) to confirm pepsin resistance, which was confirmed by simulated intestinal fluid (pH 7 and 8; adjusted with 1 N NaOH) containing 1 g/L pancreatin (Sigma-Aldrich). Mixed cells were incubated at 37 °C for 0, 1, 2, and 3 h, or 0, 1, 3, and 5 h. Under all conditions, the selected strain resistance was evaluated as cell viability using cell counts in MRS agar plates to obtain clustering units per mL (CFU/mL).

For determining bile tolerance, the strains were suspended in MRS broth containing 0–0.5% (*w*/*v*) bile salts (Oxgall; Sigma-aldrich) and incubated at 37 °C for 4 h. After incubation, cell viability was evaluated based on the cell counts on MRS agar plates, and the number of colonies was calculated as log CFU/mL.

#### 2.7.2. Auto-Aggregation Assay

Auto-aggregation assays were conducted by modifying the method of Kassaa et al. [17] to indirectly verify the attachment ability of intestinal cells. The selected strains were grown at 37 °C for 18 h in MRS broth. The cells were harvested by centrifugation at 3470× *g* for 10 min and washed twice with PBS (pH 7.0). The absorbance was adjusted to 1.0, at a wavelength of 600 nm, to standardize the number of bacteria (10^7^–10^8^ CFU/mL). Then, the cell suspension (4 mL) was incubated at room temperature for 5 h without agitation. Absorbance was measured at 600 nm for 0.1 mL of supernatant using a microplate reader. Auto-aggregation (%) was calculated using the following formula:

Auto-aggregation (%) = [1 − (A_5_/A_0_)] × 100 (3)

where A_5_ is the absorbance after 5 h of incubation, and A_0_ is the absorbance at time zero.

#### 2.7.3. Hemolytic and Enzymatic Activity

The hemolytic activity of the selected strain was detected by streaking onto tryptic soy agar (Difco) plates supplemented with 5% sheep blood and incubation at 37 °C for 48 h. The plate was then observed for verification of hemolytic patterns that appeared as a clean zone (β-hemolysis), a greenish zone (α-hemolysis), or no such zone (γ-hemolysis). Furthermore, to measure enzyme activities and carbohydrate availability, the four selected strains were grown on MRS agar plates for 18 h at 37 °C or 42 °C. The strains were assayed using API ZYM kits with cell colonies according to the manufacturer’s instructions (BioMérieux, Marcy-l’Étoile, France). Enzyme activity was determined according to coloration intensity.

#### 2.7.4. Antibiotic Susceptibility Assay

Antibiotic susceptibility of the selected strains was measured using the minimum inhibitory concentration (MIC) test strip method according to the European Food Safety Authority (EFSA) guidelines [18]. The culture solution, adjusted to a McFarland turbidity of 0.5, was spread onto Brain Heart Infusion (Difco) agar. Next, MIC test strips (Liofilchem, Teramo, Italy) were placed on the agar surface according to the manufacturer’s recommendations. The plates were incubated at 37 °C for 20 h. Nine antibiotic types were used in this test: ampicillin, chloramphenicol, clindamycin, erythromycin, gentamicin, kanamycin, streptomycin, tetracycline, and vancomycin.

### 2.8. Statistical Analysis

Statistical analysis was performed to evaluate the statistical differences using GraphPad Prism (GraphPad Prism 5.0; GraphPad Software Inc., San Diego, CA, USA). All experiments were performed in triplicate, and the results are presented as the mean ± standard error of the mean (SEM). Significant differences between the results were evaluated using Dunnett’s test.

## 3. Results and Discussion

### 3.1. Isolation and Antioxidant Effect of Candidate Strains

**Figure 1:**
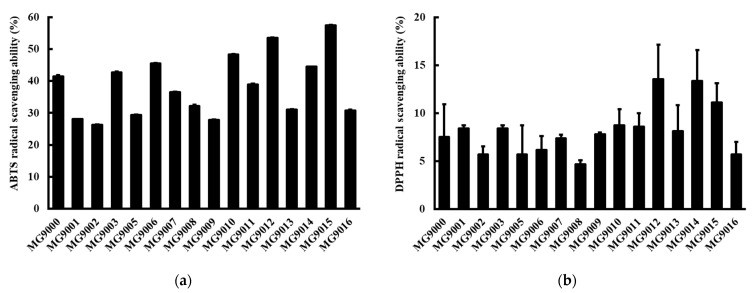
ABTS and DPPH radical scavenging activities of 16 isolated bacterial strains. (**a**) ABTS radical scavenging activity of 16 bacterial isolates; (**b**) DPPH radical scavenging activity of 16 bacterial isolates. Values represent the mean ± SEM (*n* = 3).

Sixteen isolated strains were acquired from the feces of healthy dogs and cats. The strains were identified as three species belonging to the *Pediococcus*, *Lactobacillus*, and *Enterococcus* genera, including one *P. pentosaceus* strain, four *P. acidilactici* strains, one *L. animalis* strain, two *L. plantarum* strains, two *L. fermentum* strains, one *L. reuteri* strain, and five *E. faecium* strains. Hydroxyl and related radicals are the most harmful ROS, which results in oxidative injury of biomolecules. DPPH and ABTS accept electrons or hydrogen atoms from antioxidant substances and convert them into irreversibly stable molecules [19]. The antioxidant activities of the 16 isolated strains were evaluated by DPPH and ABTS radical scavenging activities. The ABTS free radical scavenging activity of isolated strains range from 26.3 to 57.4% (Figure 1a), and *P. pentosaceus* MG9015 showed the highest activity (57.4 ± 0.2%). The DPPH radical scavenging activities of the strains range from 4.7 to 13.5% (Figure 1b), and *L. reuteri* MG9012 showed the highest activity (13.5 ± 2.7%).

**Figure 2:**
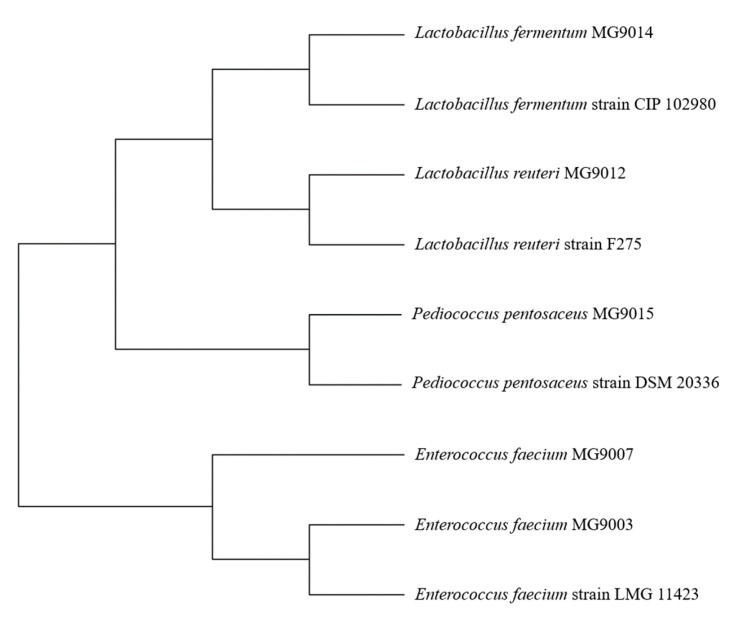
Phylogenetic analysis of selected strain based on bacterial 16S rRNA gene sequences.

Based on the DPPH and ABTS assay results, five strains (*E. faecium* MG9003(YH9003), *E. faecium* MG9007(YH9007), *L. reuteri* MG9012(YH9012), *L. fermentum* MG9014(YH9014), and *P. pentosaceus* MG9015(YH9015)) were adopted as candidates for subsequent experiments (Figure 2), considering the antioxidant effect and fermentation production (>1 × 10^9^ CFU/g) yield (data not shown) among the 16 isolated strains.

**Figure 3:**
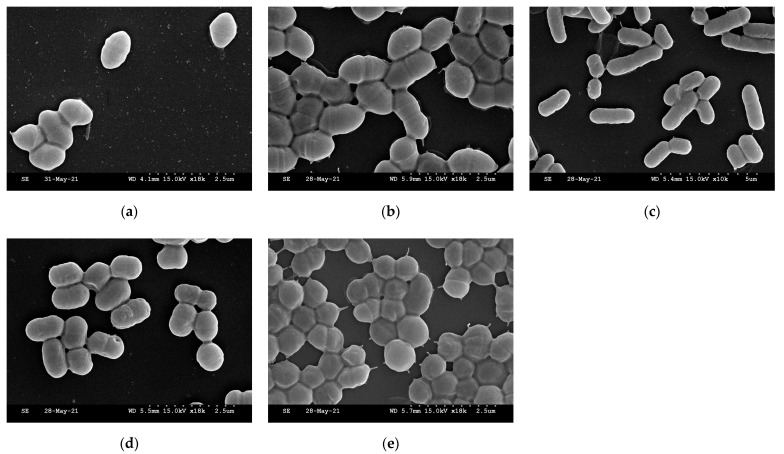
SEM images of selected strains. (**a**) *E. faecium* MG9003; (**b**) *E. faecium* MG9007; (**c**) *L. reuteri* MG9012; (**d**) *L. fermentum* MG9014; (**e**) *P. pentosaceus* MG9015.

The selected strains were identified as regular rods and include shorter forms and extended cells (Figure 3). Probiotics are known to modulate the redox state of hosts through chelating capabilities, bioactive compound production, antioxidant enzymatic systems, and antioxidant signaling pathways, and they protect hosts from oxidative stress through specific molecular mechanisms.

### 3.2. Anti-Inflammatory Effects of Selected Strains

#### 3.2.1. Inhibitory Effect by Selected Strains against NO Production

**Figure 4:**
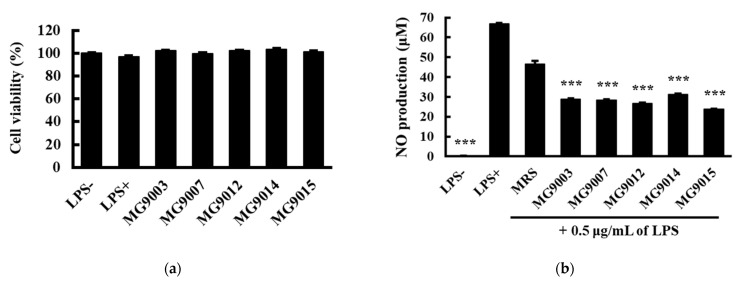

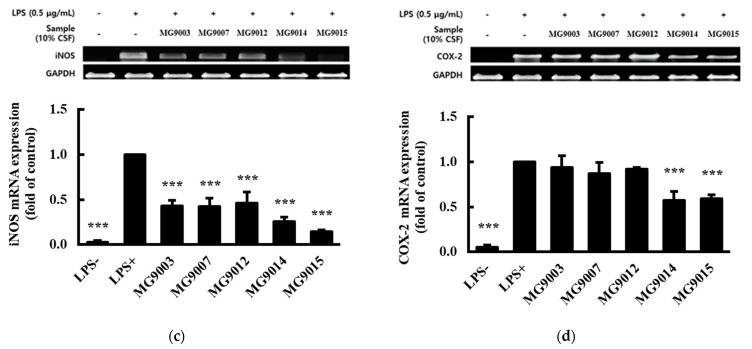
Effects of cell-free supernatants (CFSs) on cell viability, nitric oxide (NO) production, and iNOS and COX-2 expression. (**a**) Measurement of cell viability through MTT assay. (**b**) Inhibition of NO production in LPS-induced RAW 264.7 cells treated with 10% (*v*/*v*) CFSs of selected strains. Levels of (**c**) iNOS and (**d**) COX-2 mRNA expression were determined by RT-PCR. Glyceraldehyde-3-phosphate dehydrogenase (GAPDH) was used as a housekeeping gene to quantify relatively all groups. Values represent the mean ± SEM (*n* = 3). MG9003: *E. faecium*, MG9007: *E. faecium*, MG9012: *L. reuteri*, MG9014: *L. fermentum*, MG9015: *P. pentosaceus*. *** *p* < 0.001, compared to the LPS treated group (LPS+), indicates statistical significance.

The MTT assay using RAW 264.7 cells showed that the selected strains were non-toxic with cell viability of approximately 97.13–103.35%. (Figure 4a). LPS markedly induced NO production (66.9 ± 0.3 μM) compared to the control (0.2 ± 0.1 μM; Figure 4b). After 10% CFS treatment, NO production was significantly reduced in all strains (*p* < 0.001). *P. pentosaceus* MG9015 exhibited the highest NO inhibition (23.9 ± 0.1 μM) in LPS-induced cells, followed by *L. reuteri* MG9012 (26.6 ± 0.5 μM), *E. faecium* MG9007 (28.5 ± 0.2 μM), MG9003 (28.8 ± 0.3 μM), and *L. fermentum* MG9014 (31.2 ± 0.4 μM), and the inhibition rates are 64.3, 60.2, 57.4, and 53.4%, respectively (Figure 4b). In order to confirm the effect of the culture medium, the MRS group served as a positive control, and the inhibition rate of 46.5% was observed to confirm the NO production inhibitory effect of the selected strain. NO is an intracellular and intercellular signaling molecule that is derived from iNOS and forms an immune response, and it is recognized as one of the most versatile substances produced by numerous immune system cells as toxic defense molecules against infectious organisms [20]. Excessive NO production has been reported to deepen inflammatory reactions or cause genetic mutations and tissue and nerve damage, which can have fatal consequences for the host [21,22]. Therefore, inflammatory reaction regulation is an important factor in biological maintenance, and anti-inflammatory effects can be confirmed through the inhibitory effects of NO production. Therefore, the selected strains demonstrated their potential as anti-inflammatory agents that produce antioxidants and inhibit NO production.

#### 3.2.2. Anti-Inflammatory Activity of Selected Strains on RAW 264.7 Cells via Comparison of RNA Expression Levels

LPS is present in the outer membrane of Gram-negative bacteria, activating the intracellular transcription factor, NF-κB, in macrophages. It induced iNOS and COX-2 expression, which regulates pro-inflammatory mediators and NO production [23]. NO overproduction can lead to various diseases, chronic inflammation, neurodegenerative diseases, and cancer progression. Therefore, inhibiting the expression of these enzymes can affect their proinflammatory activity. RT-PCR was used to investigate the inhibitory activity of the selected strains on iNOS and COX-2 expression. The results show that the iNOS (Figure 4b) and COX-2 expression levels (Figure 4c) were increased in LPS-induced RAW264.7 cells and were inhibited by treatment with CFSs of selected strains. Additionally, iNOS expression was inhibited by the CFSs of all selected strains, while COX-2 expression was only inhibited by CFSs of the MG9014 and MG9015 strains. Apart from the mRNA transcription level, COX-2 could be controlled at the enzymatic activity level; therefore, an enzymatic activity evaluation that directly affects NO production is required in further studies. Inflammatory cell damage is mediated by oxygen-induced free radicals and high-energy oxidation, which cause toxic oxidation reactions in cells [24]. Therefore, in vitro results show that the selected strains could be applied to mitigate the effects of inflammatory diseases associated with oxidative stress and directly alleviate the factors involved in inflammatory reactions.

### 3.3. Functional Characterization of Selected Strains as Potential Probiotics

#### 3.3.1. Survival of the Strains under Simulated Gastrointestinal Conditions

**Figure 5:**
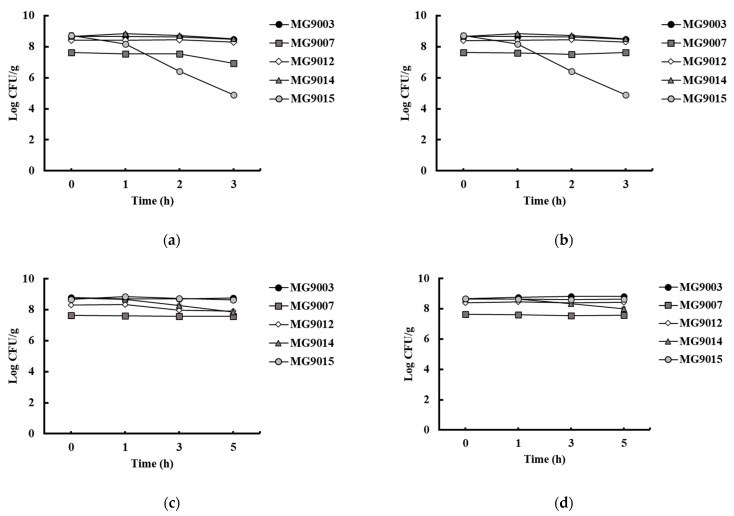
Survivability of selected bacterial strains under simulated gastrointestinal tract conditions. (**a**) Simulated gastric fluid at pH 3, (**b**) simulated gastric fluid at pH 4, (**c**) simulated intestinal fluid at pH 7, (**d**) simulated intestinal fluid at pH 8. Values represent the mean ± SEM (*n* = 3). MG9003: *E. faecium*, MG9007: *E. faecium*, MG9012: *L. reuteri*, MG9014: *L. fermentum*, MG9015: *P. pentosaceus*.

The probiotic strains were exposed to simulated gastric fluid conditions (pH 3 and 4) and intestinal conditions (pH 7 and 8). All selected strains show stable survival rates without a dramatic decrease in intestinal conditions (Figure 5). *E. faecium* MG9003 showed relatively prominent resistance among the strains and was above 8.4 log CFU/mL under intestinal conditions. In gastric conditions of pH 3 and 4, *L. fermentum* MG9015 decreased from 8.7 log CFU/mL to 4.8 log CFU/mL after 2 h of incubation; however, other strains showed high resistance without a dramatic decrease. Taken together, the selected strains were likely to survive in both the stomach and intestinal fluid. F_1_–F_0_–ATPases capable of hydrolyzing or synthesizing intracellular ATP play a role in transporting protons through the F_0_ complex, which is positively associated with acid tolerance in LAB [25]. This suggests that the selected strain exhibited high intracellular ATPase activity. LAB acid resistance has been reported to be related to changes in its glycolytic flux, its ability to control intracellular pH, and cell membrane ATPase. This suggests that the high resistance of *E. faecium* MG9003 to gastric acid was due to intracellular pH regulation and the action of an ATPase [26]. For *E. faecium* MG9007, microencapsulation technology would be a good solution for enhancing its survivability under gastric conditions [27].

After passing through the stomach, probiotic bacteria are exposed to bile salt stress. In general, microorganisms that can survive at concentrations of up to 0.3% bile salts are reported to be resistant to bile salts [28], and various species of LAB produce bile salt hydrolase to assist colonization in the host’s gut [29]. Therefore, the cell viability of selected strains in the presence of bile salts was evaluated. The number of colonies of the selected strains under bile salt stress condition decreased as the bile salt concentration increased from 0 to 1%, but all strain exhibited a viable cell count ranging from 8.32 to 9.04 log CFU/mL at a 0.5% bile salt (Table 1). Therefore, this suggests that selected strains with bile salt resistance can survive in human intestinal environments.

#### 3.3.2. Auto-Aggregation

The adherence abilities of the five probiotic strains were measured by their auto-aggregation in 5 h. All strains exhibit auto-aggregation percentages >50%, and *L. reuteri* MG9012 presented the highest auto-aggregation (63.4%) compared to the other strains (Table 2). In the present study, our strains showed relatively high auto-aggregation values [30,31,32]. The high auto-aggregation indicates that the strains possess a high potential ability to adhere to epithelial cells and mucosal surfaces [33]. Additionally, auto-aggregation is an important probiotic property in the prevention of surface colonization by pathogens [34], and LAB generally have a variable range of auto-aggregation capacities [35].

#### 3.3.3. Hemolytic Activity

Hemolysis remains the main virulence factor of pathogenic bacteria, and probiotic strains must be safe, especially within the host body. All strains exhibited the α-hemolysis effect after 48 h of incubation on blood agar plates. A previous study showed that α-hemolytic non-enterococcal lactic acid bacteria isolated from dairy products have been considered safe [36,37]. Therefore, the selected strains may have low virulence potential and could be safe for use as animal probiotics.

#### 3.3.4. Assessment of Enzyme Production

The API ZYM system is used to evaluate the enzymatic activity patterns of the selected strains (Table 3). Patterns of harmful enzyme inhibition and useful enzyme production exhibit different characteristics depending on the microorganism species; thus, the exclusion of probiotics that produce potentially toxic substances is important. The selected strains did not produce lipase, β-glucuronidase, N-acetyl-β-glucosaminidase, or α-mannosidase. Among them, β-glucuronidase is a bacterial carcinogenic enzyme that exerts negative effects on the liver [38].

#### 3.3.5. Antibiotic Susceptibility

The antibiotic resistance of the five probiotic strains was assessed using the MIC test. Results from all five strains are within the epidemiological cut-off values suggested by the EFSA [18]. *L. fermentum* MG9014 is resistant to tetracycline, and *P. pentosaceus* MG9015 is resistant to kanamycin, tetracycline, and chloramphenicol. The other three strains are sensitive to ampicillin, gentamicin, streptomycin, erythromycin, vancomycin, and clindamycin (Table 4).

LAB have been recognized as safe through “generally recognized as safe” and “qualified presumption of safety” granted by the FDA and EFSA authorities. However, it has recently been confirmed that certain LAB strains isolated from fermented foods contain genes that are resistant to erythromycin, tetracycline, and vancomycin. If antibiotic-resistant LAB are continuously exposed to environmental conditions, LAB can be a resistant or external storage space for antibiotic-resistant genes, raising concerns that they could be transmitted to pathogens through horizontal gene transfer. However, antibiotics used for therapeutic purposes can impair the functionality of probiotics. Resistance to antibiotics is important, because the use of antibiotic-resistant microorganisms can be useful in individuals with intestinal microbial imbalance caused by various antibiotics. Therefore, probiotics have a two-sided effect, and in terms of the potential risk of probiotics, the US FDA evaluated the “safety of probiotic use” with “reasonable certainty” [39].

## 4. Conclusions

This study was performed to evaluate the antioxidant and anti-inflammatory activity and identify the probiotic properties of novel LAB strains derived from animal hosts. In this study, five probiotic strains (*E. faecium* MG9003(YH9003), *E. faecium* MG9007(YH9007), *L. reuteri* MG9012(YH9012), *L. fermentum* MG9014(YH9014), and *P. pentosaceus* MG9015(YH9015)) were observed to exhibit high antioxidant activity using ABTS and DPPH, and we noted high productivity among 16 LAB strains isolated from canines and felines. The selected strains have the ability to inhibit NO production and anti-inflammatory activity via inhibition of iNOS and COX-2 gene expression. Additionally, the probiotic properties with high stability and safety under simulated gastrointestinal conditions were evaluated. The results of the current study suggest that the selected strains can be a good resource in the field of animal probiotics because they have host specificity. However, the safety and effectiveness of in vivo studies must be confirmed for further use.

## Figures and Tables

**Table 1:** Survival of the selected strains under 0.5% bile salt stress conditions.

Strains	Viable Cell Count (Log CFU/mL)		
	0 h	2 h	4 h
MG9003	8.72 ± 0.12	8.36 ± 0.01	8.32 ± 0.02
MG9007	9.04 ± 0.01	8.97 ± 0.04	8.95 ± 0.01
MG9012	8.57 ± 0.03	8.57 ± 0.05	8.56 ± 0.02
MG9014	8.92 ± 0.09	8.91 ± 0.05	8.90 ± 0.10
MG9015	8.54 ± 0.14	8.51 ± 0.11	8.51 ± 0.02

Tolerance to simulated bile salt stress conditions was evaluated based on the viable cell counts (log CFU/mL) of each strain after 4 h of incubation at 37 °C. Values represent the mean ± SEM (*n* = 3). MG9003: *E. faecium*, MG9007: *E. faecium*, MG9012: *L. reuteri*, MG9014: *L. fermentum*, MG9015: *P. pentosaceus*.

**Table 2:** The auto-aggregation of the selected strains.

Strains	Species	Origin	Auto-Aggregation (%)
MG9003	*E. faecium*	Feline	50.5
MG9007	*E. faecium*	Canine	50.0
MG9012	*L. reuteri*	Feline	63.4
MG9014	*L. fermentum*	Feline	61.0
MG9015	*P. pentosaceus*	Feline	57.7

**Table 3:** Enzyme activities of selected strains assayed using API ZYM test.

Enzyme Assayed for	Substrate	MG9003	MG9007	MG9012	MG9014	MG9015
Control (negative)	-	0	0	0	0	0
Alkaline phosphatase	2-naphthyl phosphate	0	0	0	0	0
Esterase (C4)	2-naphthyl butyrate	3	3	2	1	0
Esterase lipase (C8)	2-naphthyl caprylate	1	2	1	1	0
Lipase (C14)	2-naphthyl myristate	0	0	0	0	0
Leucine arylamidase	L-leucyl-2-naphthylamide	2	3	2	3	3
Valine arylamidase	L-valyl-2-naphthylamide	0	1	0	0	2
Crystine arylamidase	L-cystyl-2-naphthylamide	0	2	0	0	0
Trypsin	N-benzoyl-DL-arginine-2-naphthylamide	0	0	0	0	0
α-chymotrypsin	N-glutaryl-phenylanine-2-naphthylamide	0	0	0	0	0
Acid phosphatase	2-naphtyl phosphate	1	2	3	0	1
Naphtol-AS-BI-phosphohydrolase	Naphthol-AS-BI-phosphate	1	2	0	0	2
α-galactosidase	6-Br-2-naphthyl-αD-galactopyranoside	1	0	5	5	0
β-galatosidase	2-naphthyl-βD-galactopyranoside	1	0	4	5	0
β-glucuronidase	Naphthol-AS-BI-βD-glucuronide	0	0	0	0	0
α-glucosidase	2-naphthyl-αD-glucopyranoside	0	0	3	5	0
β-glucosidase	6-Br-2-naphthyl-βD-glucopyranoside	0	0	0	0	0
N-acetyl-β-glucosaminidase	1-naphthyl-N-acetyl-βD-glucosaminide	0	0	0	0	0
α-mannosidase	6-Br-2-naphthyl-αD-mannopyranoside	0	0	0	0	0
α-fucosidase	2-naphthyl-αL-fucopyranoside	0	0	0	0	0
Alkaline phosphatase	2-naphthyl phosphate	0	0	0	0	0
Esterase (C4)	2-naphthyl butyrate	3	3	2	1	0

Enzyme activities were classified from 0 (no activity) to 5 (≥40 nM of product released) with 10 nM intervals in the API ZYM color reaction chart. MG9003: *E. faecium*, MG9007: *E. faecium*, MG9012: *L. reuteri*, MG9014: *L. fermentum*, MG9015: *P. pentosaceus*.

**Table 4:** Minimum inhibitory concentrations (MICs) of different antibiotics for the selected strains.

Antibiotic with the MIC (μg/mL)	MG9003		MG9007		MG9012		MG9014		MG9015	
	MIC	S/R	MIC	S/R	MIC	S/R	MIC	S/R	MIC	S/R
Ampicillin (AMP)	0.5	S	2	S	0.19	S	0.19	S	1.5	S
Gentamicin (GEN)	12	S	32	S	0.38	S	0.38	S	4	S
Kanamycin (K)	32	S	256	S	2	S	12	S	>256	R
Streptomycin (S)	≤128	S	128	S	3	S	6	S	48	S
Tetracycline (TE)	0.125	S	2	S	2	S	>256	R	>256	R
Chloramphenicol (C)	≤6	S	8	S	1.5	S	3	S	6	R
Erythromycin (E)	2	S	2	S	0.016	S	0.032	S	0.19	S
Vancomycin (VA)	2	S	2	S	>256	n.r.	48	n.r.	>256	n.r.
Clindamycin (CD)	0.094	S	4	S	0.016	S	0.016	S	0.032	S
Ampicillin (AMP)	0.5	S	2	S	0.19	S	0.19	S	1.5	S
Gentamicin (GEN)	12	S	32	S	0.38	S	0.38	S	4	S
Kanamycin (K)	32	S	256	S	2	S	12	S	>256	R
Streptomycin (S)	≤128	S	128	S	3	S	6	S	48	S
Tetracycline (TE)	0.125	S	2	S	2	S	>256	R	>256	R
Chloramphenicol (C)	≤6	S	8	S	1.5	S	3	S	6	R
Erythromycin (E)	2	S	2	S	0.016	S	0.032	S	0.19	S
Vancomycin (VA)	2	S	2	S	>256	n.r.	48	n.r.	>256	n.r.
Clindamycin (CD)	0.094	S	4	S	0.016	S	0.016	S	0.032	S
Ampicillin (AMP)	0.5	S	2	S	0.19	S	0.19	S	1.5	S
Gentamicin (GEN)	12	S	32	S	0.38	S	0.38	S	4	S
Kanamycin (K)	32	S	256	S	2	S	12	S	>256	R

The MIC breakpoints were chosen as suggested by EFSA (2018). S: susceptible, R: resistant, n.r.: not required. MG9003: *E. faecium*, MG9007: *E. faecium*, MG9012: *L. reuteri*, MG9014: *L. fermentum*, MG9015: *P. pentosaceus.*

## Data Availability

All data are presented in the paper.
